# Pika Gut May Select for Rare but Diverse Environmental Bacteria

**DOI:** 10.3389/fmicb.2016.01269

**Published:** 2016-08-17

**Authors:** Huan Li, Tongtong Li, Minjie Yao, Jiabao Li, Shiheng Zhang, Stephan Wirth, Weidong Cao, Qiang Lin, Xiangzhen Li

**Affiliations:** ^1^Key Laboratory of Environmental and Applied Microbiology, Environmental Microbiology Key Laboratory of Sichuan Province, Chengdu Institute of Biology, Chinese Academy of SciencesSichuan, China; ^2^University of Chinese Academy of SciencesBeijing, China; ^3^Leibniz-Center for Agricultural Landscape Research (ZALF), Institute of Landscape BiogeochemistryMüncheberg, Germany; ^4^Key Laboratory of Plant Nutrition and Fertilizer, Ministry of Agriculture/Institute of Agricultural Resources and Regional Planning, Chinese Academy of Agricultural SciencesBeijing, China; ^5^Soil and Fertilizer Institute, Qinghai Academy of Agriculture and Forestry Sciences, Qinghai UniversityXining, China

**Keywords:** pikas, gut bacteriome, environmental bacteria, low abundance, diverse

## Abstract

The composition of the mammalian gut bacterial communities can be influenced by the introduction of environmental bacteria in their respective habitats. However, there are no extensive studies examining the interactions between environmental bacteriome and gut bacteriome in wild mammals. Here, we explored the relationship between the gut bacterial communities of pika (*Ochotona* spp.) and the related environmental bacteria across host species and altitudinal sites using 16S rRNA gene sequencing. Plateau pikas (*O. curzoniae*) and Daurian pikas (*O. daurica*) were sampled at five different sites, and plant and soil samples were collected at each site as well. Our data indicated that Plateau pikas and Daurian pikas had distinct bacterial communities. The pika, plant and soil bacterial communities were also distinct. Very little overlap occurred in the pika core bacteria and the most abundant environmental bacteria. The shared OTUs between pikas and environments were present in the environment at relatively low abundance, whereas they were affiliated with diverse bacterial taxa. These results suggested that the pika gut may mainly select for low-abundance but diverse environmental bacteria in a host species-specific manner.

## Introduction

Host-microbe symbioses are widespread and ubiquitous in humans and animals. The symbiotic microbial communities may help hosts achieve many critical physiological functions, such as food digestion and energy harvest (Tremaroli and Backhed, 2012), and immune regulation (Zhang et al., 2015).

After birth, the acquisition of symbiotic microbes by mammalian hosts is mainly achieved via two modes: vertical transmission (host acquires microbes from parents), and horizontal transmission (microbes obtained from environment or non-parent conspecifics) (Inoue and Ushida, 2003). In some host-microbe symbioses, the vertical transmission plays a major role in the assembly of microbial communities. The symbiotic microbes in insects (Hosokawa et al., 2007; Damiani et al., 2008) or rats (Inoue and Ushida, 2003) are primarily vertically transmitted. However, in other systems, host-associated symbionts may predominantly be acquired from the environment in each new generation. For example, this mode of transmission often occurs in the stinkbug-*Burkholderia* (Kikuchi et al., 2007) and the squid-*Vibrio* (Nyholm and McFall-Ngai, 2004) symbioses. Notably, many animals, including amphibians (Walke et al., 2014) and sponges (Sipkema et al., 2015), rely on a combination of vertical transmission and horizontal transmission of symbiotic microbes.

Understanding the modes of microbial transmission for a given host is important, because these symbionts may strongly influence host ecology. For instance, socially-transmitted bacteria through horizontal transmission may confer resistance to natural enemies (Moran and Dunbar, 2006; Jaenike et al., 2010; Łukasik et al., 2013), improve the utilization efficiency of new diet resources (Tsuchida et al., 2004), and increase tolerance to extreme environment (Montllor et al., 2002). In addition, the spread or acquisition of these microbial symbionts may also influence evolutionary patterns in some systems. For example, symbionts can undergo strikingly convergent patterns of genome reduction through strict vertical transmission (Moran et al., 2008). The resulting small genomes exhibit vast AT nucleotide enrichment, and thus undergo accelerated molecular evolution (Shigenobu et al., 2000; McCutcheon and Moran, 2007). Due to gene loss, some specified functional roles of these symbionts may be limited. In contrast, symbionts reliant on horizontal transmission generally have large, expanded genomes, and horizontal transmission promotes genetic exchanges between host-associated microbes and environmental microbes, potentially modulating conflicts of fitness interests between hosts and symbionts (Sachs et al., 2011).

Studies on transmission in animal-microbe symbioses showed that various factors, such as host genetics (McKenzie et al., 2012), diet (Carmody et al., 2015; Dill-McFarland et al., 2016), and habitat degradation (Amato et al., 2013; Barelli et al., 2015), influence the composition of gut microbial communities. Several studies on fish, including grass carp (*Ctenopharyngodon idellus*) (Wu et al., 2012), gibel carp (*Carassius auratus gibelio*) (Wu et al., 2013), and zebrafish (*Danio rerio*) (Wong et al., 2015), suggest that gut microbial communities do not simply reflect those microbes presented in their surrounding environments, but hosts select for specific environmental microbes. The fish intestine consists of a common core microbial community regardless of differences in host environments (Roeselers et al., 2011; Li et al., 2015). In addition, differences in the skin-associated bacterial communities of sympatric amphibian species in the same pond environment suggest that host species-specificity, not pond environment, determines the composition of microbial communities (McKenzie et al., 2012; Walke et al., 2014). Yet to date, few studies have directly estimated the relationship between the gut bacteriome of wild mammals and the related environmental bacteriome.

Pika (*Ochotona* spp.) is a small herbivorous mammal whose range spans through Asia, America and Europe (Yang et al., 2008), while they are considered to have a common Asian origin. A majority of the Asian pikas are widely distributed on the Qinghai-Tibet Plateau and adjacent regions (Niu et al., 2004). In China, there are two common pika species, including plateau pikas and Daurian pika. These two pika species are thought to have diverge 6.5 million years (Yu et al., 1998). Plateau pika is considered as a keystone species in the Qinghai-Tibet Plateau and prefers to live at cold, hypoxic high altitude regions with generally more than 3000 m above sea level (m.a.s.l.) (Luo et al., 2008). In contrast, the Daurian pika mainly inhabits the Inner Monglia grassland and lives in relatively mild, moderate environments at lower average altitudes of 1000 m.a.s.l. on average (Liao et al., 2006). They cross over large-cale geographical areas and utilize different food resources, thus host-gut bacteriome and environmental bacteriome may show manifest variations in different habitats. Thus, it will be conducive to exploring the relationship between gut bacteria and environmental bacteria broadly. In the grassland, pikas generally consume live plants from the surrounding environments, while they have to eat soil under starvation when food is scarce. Thus it is possible that microbes are transferred from the environment (plant and soil) to pika guts. However, the relationship between the pika gut bacterial communities and the environmental bacterial communities remains unknown.

To understand the associations between the pika gut bacteriome and the related environmental bacteriome, we sequenced the pika gut bacterial communities at five different altitudinal sites (plateau pikas at three sites, Daurian pikas at two sites) as well as the bacterial communities of the surrounding environment (plant and soil) at each site. We addressed two key questions. First, we examined the composition and structure of pika, plant and soil bacterial communities in each site. Second, we tested which bacteria in the environment were more likely to colonize into the pika gut. The results of this study improve our understanding of host-microbe interactions in wild environments, especially in the context of transmission of bacterial symbionts.

## Materials and methods

### Sample collection

Samples were collected between July 13 and August 14, 2014. Wild plateau pikas were captured from three high altitude sites (4331, 3856, and 3694 m.a.s.l.) in the Qinghai-Tibet Plateau. Daurian pikas were collected from two low altitude regions (1198 and 1000 m.a.s.l.) in the Inner Mongolia grassland. The area of each of these five sampling locations was approximately 2500 square meters (50 × 50 m). A total of 102 wild pika samples were collected, including 76 plateau pikas and 26 Daurian pikas. Upon capture, we euthanized and dissected each animal. Caecal contents were immediately collected into 50 ml sterile tubes and frozen at −20°C in a portable freezer. All animal experiments were conducted in conformity with the Institution of Animal Care and the Ethics Committee of Chengdu Institute of Biology, Chinese Academy of Sciences.

In order to understand the environmental bacteriome of the pikas' habitats, we collected 47 soil samples (0–10 cm; 5–15 samples per site) from the five sites. Within each site, 5–15 plots (1 × 1 m^2^) were randomly placed, with the stipulation that the plots were at least 10 m apart. Within each plot, each sample was a mixture of 5–7 individual soil cores at the depth of 0–10 cm. In addition, 36 plant samples (4–12 samples per site) were also collected from the five sites. The dominant plant species at each site were identified according to the morphological characteristics. The plant community on the Qinghai-Tibet Plateau was dominated by *Ajuga lupulina, Carex moorcraftii, Elymus nutans, Kobresia humilis, Ligularia virgaurea, Oxytropis* sp., *Potentilla anserine, Pedicularis kansuensis, Pedicularis* sp., *Thalictrum petaloideum*, and *Tibet Lancea* (Table S1). In contrast, the plant community on the Inner Mongolia grassland was dominated by *Chenopodium acuminatum, Cleistogenes squarrosa, Convolvunlus ammanii, Kochiaprostrata sp., Potentilla tanacetifolia, Setaria viridis*, and *Stipa* sp. (Table S1). In our study, the habitat types of Plateau pikas and Daurian pikas were alpine meadow and typical steppe ecosystem, respectively. Although bacteria from other environmental substrates (for example, rare plant species) may colonize the pika gut, we only focused on these major plant species, which are probably the main diet resources for pikas. The aboveground parts (leaves and stems) of these plants were stored in 50 ml sterile tubes with sterile scissors. Each plant sample (or species) was a mixture pooled from three same plants. All the samples were transported to our laboratory in Chengdu Institute of Biology within 24 h, and stored at −40°C for bacterial community analysis. The detailed information of each sample is listed in Table S1 and Table 1.

**Table 1:** **Sample information across 185 samples described in this study**.

**Group name**	**Common name**	**Altitude(m)**	**Weight(g)**	**NO.^a^**	**Habitat**	**District**
4331_PP	Plateau pika	4331	144 ± 46	17	Alpine meadow	Qinghai-Tibet Plateau
4331_Plant	Plant	4331	NA	5	Alpine meadow	Qinghai-Tibet Plateau
4331_Soil	Soil	4331	NA	7	Alpine meadow	Qinghai-Tibet Plateau
3856_PP	Plateau pika	3856	144 ± 25	30	Alpine meadow	Qinghai-Tibet Plateau
3856_Plant	Plant	3856	NA	11	Alpine meadow	Qinghai-Tibet Plateau
3856_Soil	Soil	3856	NA	15	Alpine meadow	Qinghai-Tibet Plateau
3694_PP	Plateau pika	3694	148 ± 23	29	Alpine meadow	Qinghai-Tibet Plateau
3694_Plant	Plant	3694	NA	12	Alpine meadow	Qinghai-Tibet Plateau
3694_Soil	Soil	3694	NA	15	Alpine meadow	Qinghai-Tibet Plateau
1198_DP	Daurian pika	1198	150 ± 29	14	Typical steppe	Inner Mongolia
1198_Plant	Plant	1198	NA	4	Typical steppe	Inner Mongolia
1198_Soil	Soil	1198	NA	5	Typical steppe	Inner Mongolia
1000_DP	Daurian pika	1000	111 ± 41	12	Typical steppe	Inner Mongolia
1000_Plant	Plant	1000	NA	4	Typical steppe	Inner Mongolia
1000_Soil	Soil	1000	NA	5	Typical steppe	Inner Mongolia

a *The number of samples in each group. The number of individual animals included in each sample set*.

### DNA extraction, PCR amplification, and next-generation sequencing

Each plant sample consisted of leaves and stems. All plant samples were then ground in liquid nitrogen before DNA extraction. Thereafter, the whole-community DNA was extracted from each sample (pika, plant and soil) using Ezup Genomic DNA Extraction Kit for Soil (Sangon Biotech, China) with slight modification. 0.3 g sterile glass beads and 0.1 g ceramic beads were added to each sample, which may improve extraction quality and yield of the sample DNA (Yu and Morrison, 2004). The concentration of total DNA from each sample was estimated using a Nanodrop 2000 Spectrophotometer (Thermo Scientific, IL, USA). The protocols of PCR amplification, gel extraction and sequencing library construction were described previously (Li et al., 2016). Finally, samples were sequenced using an Illumina MiSeq sequencer (MiSeq Reagent Kit V.2, 500 cycles) at Environmental Genomic Platform of Chengdu Institute of Biology.

### Bioinformatics analysis

The raw reads were analyzed using QIIME Pipeline–Version 1.7.0 (http://qiime.org/tutorials/tutorial.html). Sequences were split based on their unique barcodes. Sequence merging, filtering and analysis were described previously (Li et al., 2016). Briefly, after filtering out low-quality sequences, chloroplasts and chimeras, all the sequences were then clustered into operational taxonomic units (OTUs) at a 97% identity threshold using CD-HIT (Li and Godzik, 2006). Because archaeal sequences only accounted for a very small fraction of total reads (<0.01%) in pika guts, and we only focused on bacterial communities. Thus, those sequences not classifying to bacteria (Eukaryota and Archaea lineages) were removed. Singleton sequences were also filtered out. Representative sequences for each OTU were picked according to the command line of QIIME script “pick_rep_set.py” (http://qiime.org/scripts/pick_rep_set.html). Thereafter, the sequences were aligned against the Greengenes 13_8 reference database (DeSantis et al., 2006) using PyNAST tool. The phylogenetic tree of representative sequences was created using the FastTree software (Price et al., 2009). Taxonomic classification of representative sequences was implemented using the Ribosomal Database Project classifier in the QIIME platform (Wang et al., 2007).

Due to different sequencing depths, all samples were rarefied to 4058 sequences per sample. Thereafter, the mean relative abundances of OTUs were calculated for each sample. The core microbes were defined as those OTUs that are present on at least 80% of samples in each pika species. In order to assess alpha diversity measures, phylogenetic diversity and observed species were calculated. To assess beta diversity, we applied principal coordinate analysis based on the unweighted and weighted UniFrac distance metrics, which use phylogenetic information to calculate community similarity (Lozupone and Knight, 2005). Principal coordinates analysis (PCoA) was performed based on unweighted and weighted UniFrac distance metrics.

The original sequence data are available at the European Nucleotide Archive by accession number PRJEB12381 (http://www.ebi.ac.uk/ena/data/view/PRJEB12381).

### Statistical analysis

Because the sample size was uneven in each group, analysis of similarity (ANOSIM) (Dill-McFarland et al., 2016) was applied to evaluate if microbial communities were significantly different across groups. ANOSIM analysis was implemented using the procedure “anosim” in the R “vegan” package (Warton et al., 2012). One-way-analysis of variance (ANOVA) was used to evaluate the difference of microbial phyla and genera across different groups using SPSS 13.0 software. The mean relative abundance of phyla and genera in pika, plant and soil bacteriome was calculated in each sample site.

Venn diagrams were created after sequences were subsampled using the program “VennDiagram” in R to visualize the OTUs that were shared between pika, plant, and soil in each sampling site and among pikas at the five sampling sites. To explore the potential relationship between pika and environmental bacteriome, we calculated the proportion of pika OTUs that were also present in the plant or soil in each sample site. Thereafter, we calculated the mean relative abundances of shared OTUs between pika and plant or soil. In addition, we also calculated the total abundances of shared OTUs between pika and plant or soil. Taxonomic classifications of all OTUs shared by pika and environment (plant and soil) were used to generate phylum-level and genus-level abundance distributions in each site. Only the average phylum-level abundance distributions in each site were used to create pie charts.

To explore the functional profiles of pika core OTUs and shared OTUs between pika and environmental bacteriome, PICRUSTv1.0.0 (Langille et al., 2013) was used to predict gene content based on OTU abundances of 16Sr RNA sequences per sample. For this analysis, OTUs were closed-reference picked against the Greengenes database according to the online tutorial. The resulting data set was rarefied to 4058 16S rRNA sequences per sample. We predicted the metagenome for pika core OTUs that presented in at least 80% of samples, as well as the metagenome for the shared OTUs between pika and environmental bacteriome. Significant differences (*t*-tests, Bonferroni-corrected) of the predicted gene functions between pika core OTUs and shared pika-environment OTUs were tested.

## Results

### Compositional and structural differences between pika gut bacteriome and environmental bacteriome

**Figure 1:**
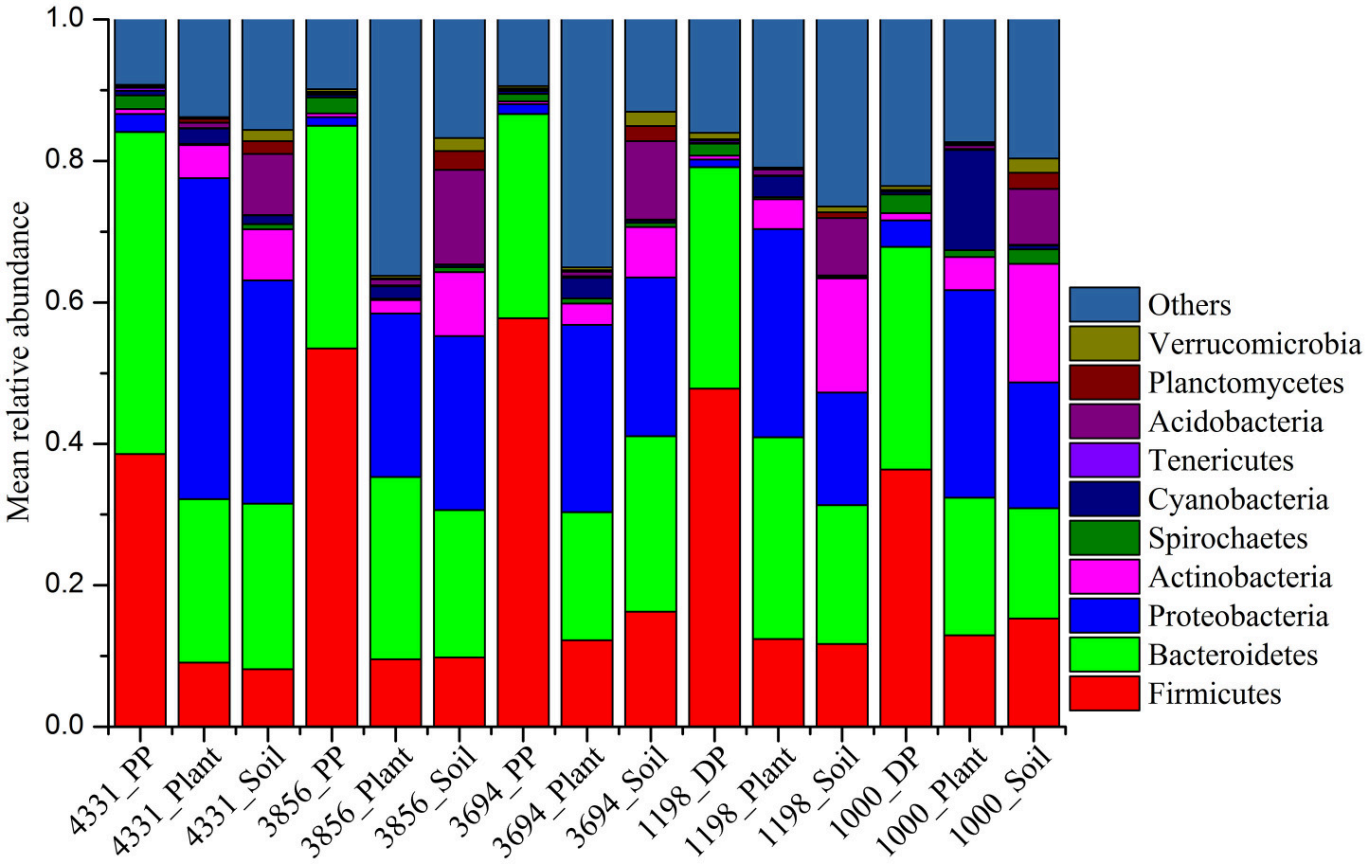
**Mean relative abundances of bacterial phyla across pikas (DP, Daurian pikas; PP, Plateau pikas), plant, and soil samples at five different sites**. Only those phyla with > 0.04% mean relative abundance across all samples are shown.

At phylum level, the pika gut bacteriome across all sites were dominated by Firmicutes and Bacteroidetes, followed by Spirochaetes and Proteobacteria (>1% relative abundance), with mean relative abundances across all pika samples of 49.4, 33.1, 1.8, and 1.8%, respectively. However, the soil bacteriome were dominated by Proteobacteria (23.3%), followed by Bacteroidetes (21.8%), Firmicutes (12.4%), Acidobacteria (10.8%), Actinobacteria (9.8%), Planctomycetes (2.1%), and Verrucomicrobia (1.8%) across all soil samples. In contrast, the plant microbial communities mainly consisted of Proteobacteria (28.7%), Bacteroidetes (22.5%), Firmicutes (11.1%), Cyanobacteria (3.7%), and Acidobacteria (3.2%). The mean relative abundance of pika, plant, and soil bacteriome at phylum level in each site was visualized in Figure 1.

**Figure 2:**
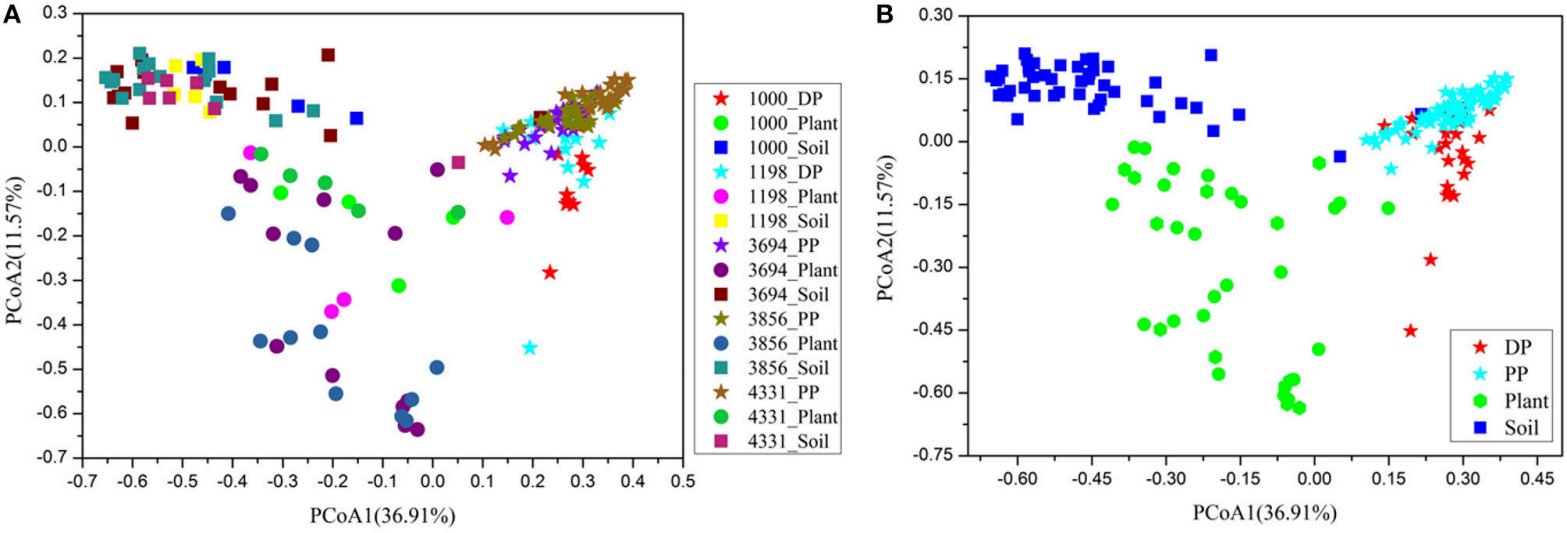
**Principal coordinates analysis (PCoA) of pika, plant and soil bacterial communities across 185 samples based on the weighted UniFrac distance metrics. (A)** 15 groups, including pika, plant, and soil bacteriome at five different altitudinal sites. **(B)** 4 groups, including DP (Daurian pikas), PP (Plateau pikas), plant and soil.

We calculated the mean phylogenetic diversity and observed species of pika, plant, and soil bacterial communities in each altitudinal site. The alpha diversity of pikas were higher than those of plant, while were lower than those of soil (Table S2). Differences in the pika, plant and soil bacterial community structure were evident (ANOSIM, *r* = 0.788, *P* < 0.001) based on the weighted UniFrac distance metrics (Figure 2). The pika gut bacteriome was more similar to the plant bacteriome than the soil bacteriome. Despite a partial overlap, the plateau pikas and Daurian pikas had distinct bacterial communities (ANOSIM, *r* = 0.411, *P* < 0.001). Sampling sites had significant impacts on the bacterial community structure of plateau pikas (ANOSIM, *r* = 0.287, *P* < 0.01) or Daurian pikas (ANOSIM, *r* = 0.24, *P* < 0.01). Results using unweighted and weighted UniFrac distance metrics were similar (Figure S1).

### Microbes that were abundant in pika guts were present in the environment at relatively low abundance

We calculated the shared and unique OTUs among pikas, plant, and soil bacteriome in each altitudinal site (Figure S2). Most gut OTUs in pikas were not observed in the environmental samples (Table S3). For example, the percentage of unique gut OTUs in Daurian pikas at 1000 and 1198 m.a.s.l. were 79% (7491 of 9473 total OTUs) and 81% (9665/11958 total OTUs), respectively. The proportion of unique gut OTUs in plateau pikas at 3694, 3856, and 4331 m.a.s.l. were 78% (14889 of 18990 total OTUs), 77% (15545 of 20121 total OTUs), and 80% (10955 of 13681 total OTUs), respectively. In particular, the proportion of shared OTUs between pika and plant were 9, 11, 9, 7, and 8% at those five sampling sites of 1000, 1198, 3694, 3856, and 4331 m.a.s.l., respectively, whereas the corresponding proportion of shared OTUs between pika and soil were 17, 12, 18, 20 and 16%, respectively. There was also overlap between plant and soil microbial communities. Lastly, pikas across the five sites shared 2703 OTUs (Figure S3). Notably, these counts of shared OTUs are sensitive to sampling effort; more extensive and even sampling of plant and soil bacteriome would possibly reveal additional microbes, and might therefore influence these estimates of overlap within the pika gut bacteriome.

Most OTUs (> 90%) that were shared between any pika host and the environment were at relative abundances of 0.1% or less in the plant or soil (Figures S4, S5, cluster of points near origin). Those OTUs that were abundant in pika guts showed a relatively low abundance in the environment (plant or soil), and the more abundant environmental OTUs had a relatively low abundance in pika gut regardless of sampling sites.

**Figure 3:**
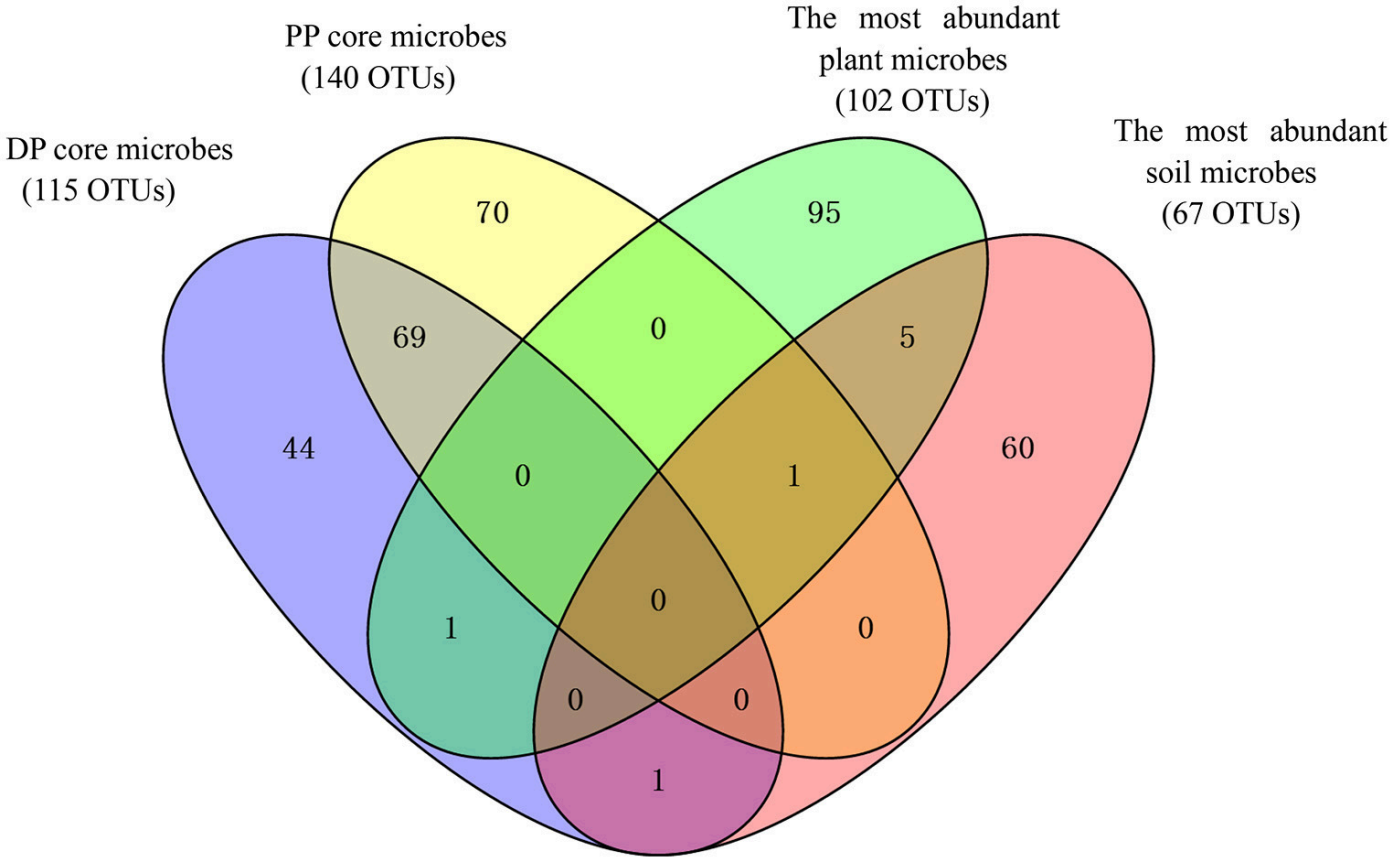
**Venn diagram showing the overlap between pika (DP, Daurian pikas; PP, Plateau pikas) core microbes and the most abundant environmental microbes (> 0.1% relative abundance)**.

The core pika gut bacterial communities were defined as those OTUs that were present on at least 80% of all individuals in each pika species. The core bacterial communities of Daurian pikas and plateau pikas included 115 and 140 OTUs, respectively (69 OTUs were shared between them). The taxonomic profiles and mean relative abundances of these OTUs were listed in Table 2. First, the total abundances of core OTUs were high in pika gut, while these OTUs had a relatively low abundance in the plant or soil bacterial communities (Table 2). Second, the majority of the pika core microbes were enriched in bacterial taxa that had low abundances (<0.1%) in the related environment. Notably, two core OTUs in Daurian pikas and three core OTUs in plateau pikas were not observed in any environmental samples, respectively. Third, most of the core OTUs (104 of 115 in Daurian pikas, and 138 of 140 in plateau pikas) were only sporadically observed in the plant bacteriome (<80% of all plant samples) in their respective environments. Lastly, we calculated 102 and 67 most abundant OTUs in plant and soil bacteriome (>0.1% relative abundance), respectively. Ninety-eight percentage of plant OTUs (100 of 102 OTUs) and 97% (65 of 67 OTUs) of soil OTUs were present in pika guts at <0.1% relative abundance. Among these environmental OTUs, 35 plant OTUs, and 2 soil OTUs were not observed in any pika samples (data are not shown), suggesting that pika gut is not well-colonized by the abundant environmental microbes. Notably, most of these dominant environmental OTUs were not the members of the pika core bacteria (Figure 3).

**Table 2:** **List of pika core OTUs (≥80% prevalence in pika populations) at five different altitude sites, and the mean relative abundances of these OTUs in pika guts and in the environments at five different altitude sites**.

**Altitude(m.a.s.l.)**		**1000**			**1198**				**3694**			**3856**			**4331**		
	**Mean relative abundances(%) of pika core OTUs**																
	**DP**	DP	Plant	Soil	DP	Plant	Soil	**PP**	PP	Plant	Soil	PP	Plant	Soil	PP	Plant	Soil
Taxonomic units	NO.^*^							NO.^*^									
Unclassified Bacteria	27	**12.94**	0.83	1.1	**7.28**	0.73	1.18	20	2.32	0.25	0.17	2.47	0.01	0.47	2.50	0.51	0.64
Bacteroidetes (**Bac**)	1	0.17	0.06	0.05	0.21	0.06	0.02	2	2.32	0.25	0.17	2.47	0.01	0.47	2.50	0.51	0.64
Bacteroidales (**Bac**)	9	2.00	0.04	0.02	1.42	0.08	0.07	18	**3.85**	0.07	0.09	**3.13**	0.04	0.14	**3.91**	0.08	0.21
*Prevotella* (**Bac**)	8	1.57	0.02	0.06	0.83	0.01	0.11	24	**3.17**	0.05	0.18	**4.07**	0.04	0.16	**6.75**	0.08	0.18
S24-7 (**Bac**)	26	**3.41**	0.45	0.39	2.74	0.31	0.26	12	1.33	0.03	0.14	1.79	0.04	0.11	2.35	0.28	0.19
Firmicutes (**Fir**)	9	**6.95**	0.02	0.39	**4.61**	0.25	0.70	8	1.79	0.02	0.07	1.61	0.00	0.27	1.36	0.04	0.22
*Streptococcus* (**Fir**)	1	0.40	0.03	0.12	0.11	0.10	0.14	1	0.12	0.02	0.02	0.12	0.00	0.05	0.12	0.03	0.10
Clostridiales (**Fir**)	19	2.88	0.42	0.52	**3.84**	0.67	0.42	32	**6.53**	0.56	0.41	**6.66**	0.16	0.40	**3.55**	0.78	0.49
Ruminococcaceae (**Fir**)	5	0.49	0.01	0.03	0.61	0.03	0.07	8	1.52	0.02	0.03	1.11	0.00	0.07	0.69	0.01	0.04
*Oscillospira* (**Fir**)	3	0.28	0.09	0.10	0.27	0.06	0.11	9	1.77	0.09	0.11	1.29	0.06	0.09	0.81	0.05	0.08
Pasteurellaceae (**Pro**)	2	0.43	0.12	0.45	0.10	0.26	0.11	1	0.05	0.01	0.01	0.07	0.00	0.04	0.05	0.16	0.15
*Treponema* (**Spi**)	3	0.92	0.02	0.16	0.46	0.04	0.03	1	0.11	0.00	0.06	0.15	0.01	0.08	0.22	0.01	0.10
*Akkermansia* (**Ver**)	2	0.36	0.14	0.19	0.57	0.06	0.05										
*YRC22* (**Bac**)								1	0.07	0.00	0.01	0.08	0.00	0.01	0.16	0.01	0.01
Cyanobacteria (**Cya**)								1	0.09	0.00	0.00	0.08	0.00	0.00	0.07	0.00	0.00
*Lactobacillus* (**Fir**)								1	0.43	0.27	0.20	0.36	0.21	0.25	0.08	0.00	0.07
*Clostridium* (**Fir**)								1	0.11	0.00	0.03	0.11	0.00	0.01	0.12	0.05	0.02
Sum	115	**32.81**	**2.26**	**3.68**	**23.05**	**2.66**	**3.28**	140	**25.57**	**1.65**	**1.69**	**25.56**	**0.57**	**2.65**	**25.23**	**2.62**	**3.11**

*DP, Daurian Pikas; PP, Plateau Pikas. m.a.s.l., meter above sea level. The lowest taxonomic resolution that could be defined for OTU identification is listed; Bacterial phyla for pika core OTUs are listed in parentheses (**Pro** = Proteobacteria, **Bac** = Bacteroidetes, **Fir** = Firmicutes, **Spi** = Spirochaetes. **Ver** = Verrucomicrobia, **Cya** = Cyanobacteria); The total mean abundance of pika core OTUs and corresponding OTUs in plant and soil is also listed. Three most abundant taxonomic units in pika guts are denoted in bold*.

* *The numbers of core OTUs belonging to the specific taxonomic unit*.

**Figure 4:**
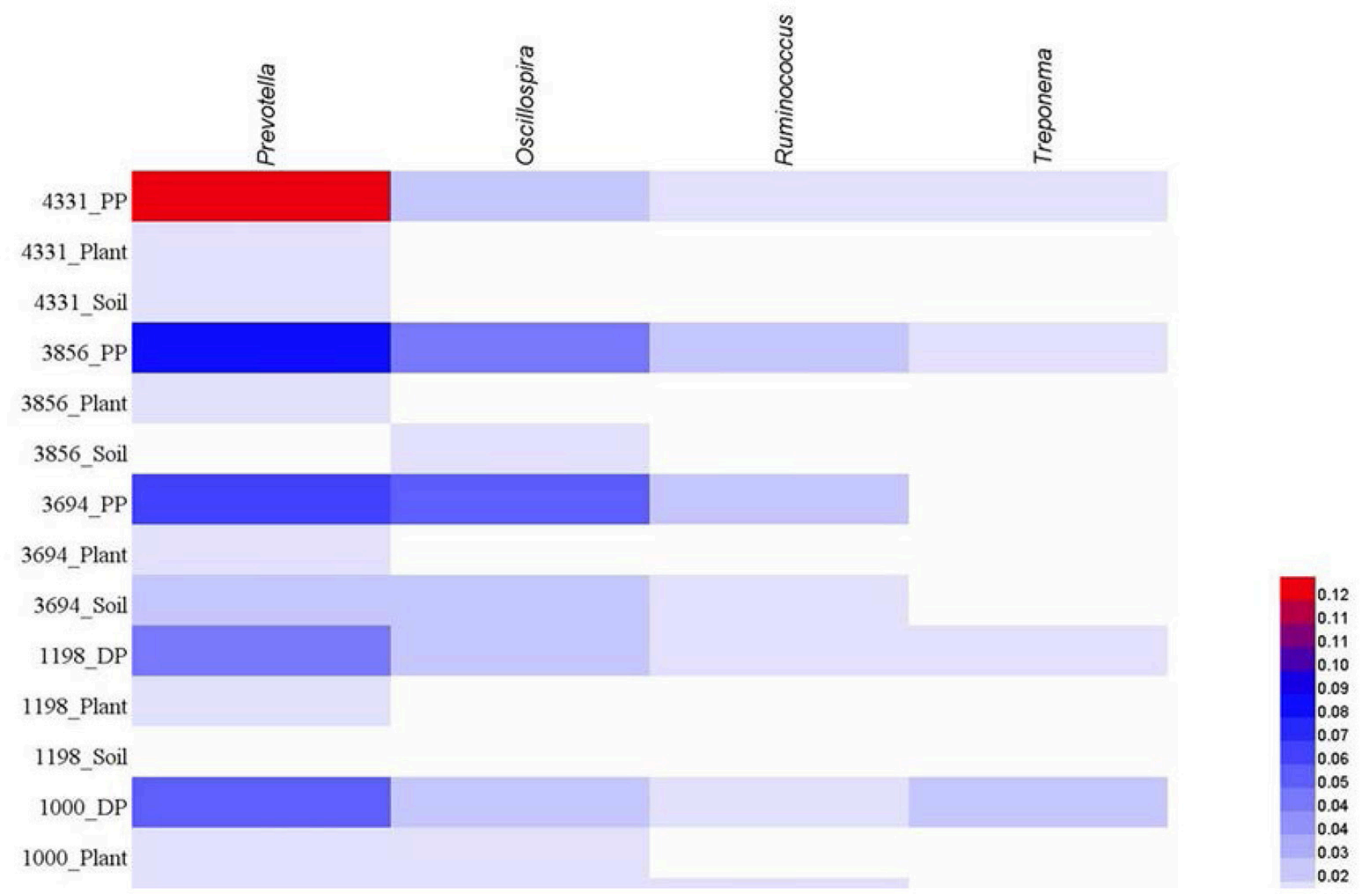
**The four dominant genera (> 1% mean relative abundance) across all pika samples (DP, Daurian pikas; PP, Plateau pikas), and corresponding abundance across plant or soil samples in each site**.

At genus level, we calculated the abundance of four most dominant genera in pika guts, including *Prevotella, Oscillospira, Ruminococcus, Treponema*, with mean relative abundance across all pika samples of 7.7, 3.8, 2.0, and 1.5%, respectively. The mean abundances of these four genera in soil bacteriome were 1.5, 1.5, 1.0, and 0.3%, respectively, and in plant bacteriome were 1.3, 0.7, 0.7, and 0.2%. The mean relative abundances of these four genera in each sample site were visualized in Figure 4. Similarly, these dominant genera in pika guts occurred at relatively low abundance in the environment.

### The shared OTUs between pika and environment represent diverse microbial taxa

To understand which microbes can be transmitted from environments to pika, we calculated the taxonomic composition of shared OTUs between pika and environment using pika samples from each altitudinal site. Despite a little difference across sites, the major phyla (>1% average relative abundance) of these shared OTUs all included Firmicutes, Bacteroidetes, Proteobacteria and Spirochaetes in each site (Figure S6). At genus level, *Prevotella, Oscillospira, Ruminococcus, Treponema* (>1% average relative abundance) were dominant based on the taxonomic composition of the shared OTUs, and their relative abundances across pikas were presented in Table S4. Other rare genera (<1% average relative abundance), such as *Streptococcus, YRC22, Lactobacillus, Phormidium* and *Coprococcus*, were shared between pika and environments.

### Functional prediction of the shared OTUs between pika and environments

**Figure 5:**
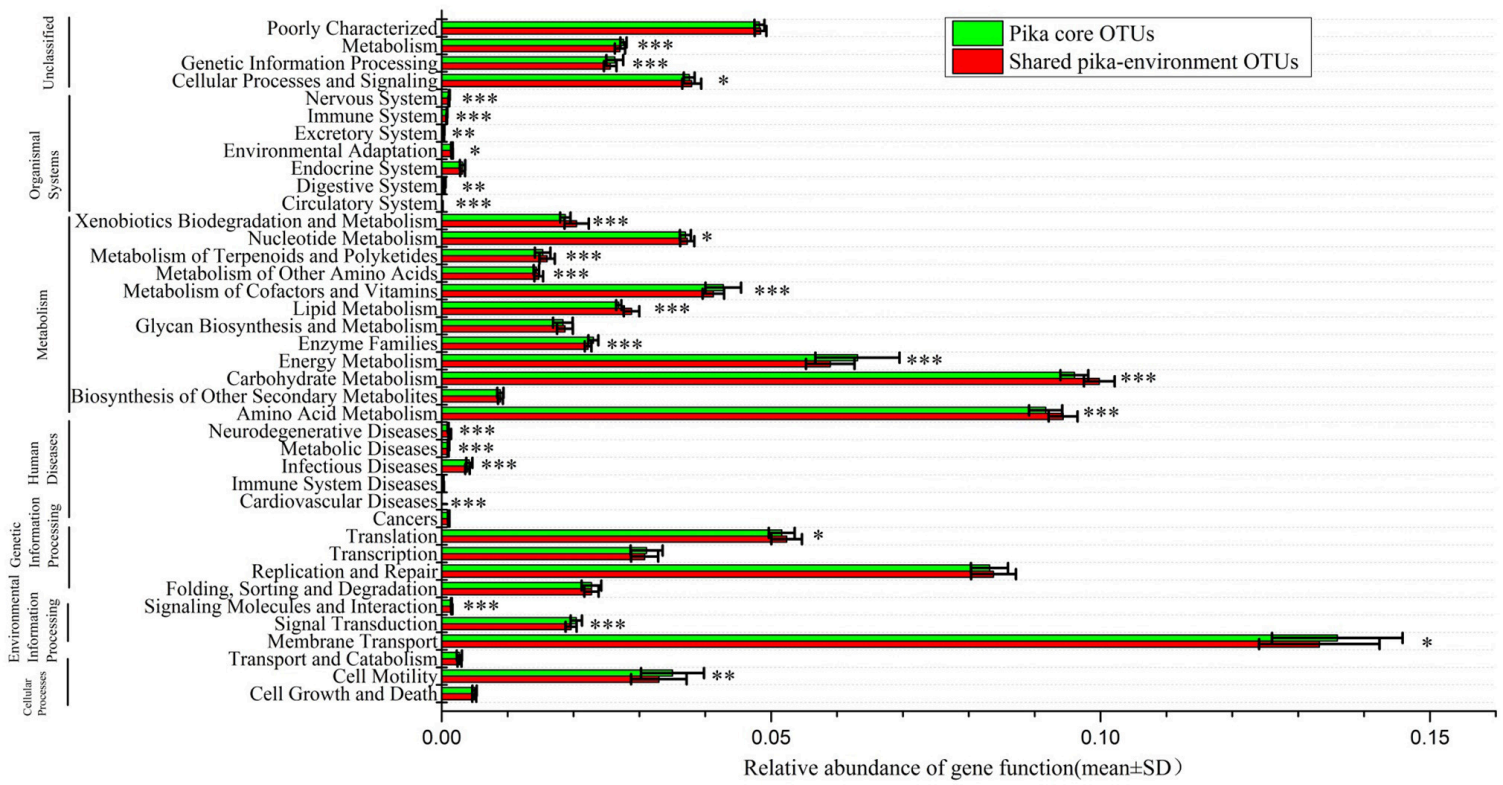
**Predicted gene functions of shared pika-environment OTUs and pika core OTUs across all samples based on the KEGG Orthology groups (KOs) at level 2**. The significance is indicated by ^*^P < 0.05; ^**^P < 0.01; ^***^P < 0.001.

To understand the function profiles of shared microbes between pika and environment, PICRUSt was used to predict the gene family and content based on their shared OTUs. Using KEEG database, we found that a total of 40 gene functions at level 2 were present in our data sets. Those genes involved in membrane transport, carbohydrate metabolism and amino acid metabolism were dominant in the whole functional profile. When compared to the gene function of pika core bacteria, 28 gene families showed statistically significant differences (*t*-tests, Bonferroni-corrected *P* < 0.05) between the shared pika-environment OTUs and pika core OTUs (Figure 5). We found that that following gene functions mainly associated with metabolism were overrepresented in the shared bacteria compared to those in the core bacteria, including carbohydrate metabolism, amino acid metabolism, metabolism of other amino acids, lipid metabolism, metabolism of terpenoids, and polyketides, nucleotide metabolism and xenobiotics biodegradation and metabolism. In particular, we illustrated the significant differences in carbohydrate metabolism. The relative abundances of ascorbate and aldarate metabolism, butanoate metabolism, citrate cycle (TCA cycle), fructose, and mannose metabolism, glycolysis/gluconeogenesis, glyoxylate, and dicarboxylate metabolism, inositol phosphate metabolism, pentose and glucuronate interconversions, Pentose phosphate pathway, propanoate metabolism, pyruvate metabolism and starch, and sucrose metabolism tended to be higher in the shared pika-environment OTUs compared to the core pika bacteria (Figure S7).

## Discussion

### The divergence between pika gut bacteriome and environmental bacteriome

The two most abundant phyla in pika guts identified in this study (Firmicutes and Bacteroidetes) accounted for more than 80% of 16S rRNA gene sequences. These results were consistent with the dominant groups found in the guts of other mammals, such as rabbits (Bäuerl et al., 2014), house mice (Linnenbrink et al., 2013), and humans (Lozupone et al., 2012). Firmicutes and Bacteroidetes are mainly responsible for food fermentation in the gut (Ley et al., 2006). However, two most dominant phyla in environment (plant or soil) were Proteobacteria and Bacteroidetes, indicating an obvious difference of gut bacterial community composition between pikas and environments.

Although plateau pikas and Daurian pikas are derived from a common ancestor, these two pika populations had distinct habitat types, diet resources, and host genetics. All of these factors may contribute to the divergence of gut bacteriome. We found the composition and structure of pika gut bacteriome were more similar to each other than environmental bacteriome regardless of geographical location, indicating that host factors are more important than environmental factors in shaping gut bacteriome. Thus, even though environmental microbes can flow into the host intestine by ingested food, pika gut microbial communities are not determined solely by passive inoculation. Rather, host factors appear to select for and maintain the gut bacteriome at similar composition and structure regardless of geographical location or habitat. Correspondingly, the same patterns have been found in amphibians (Loudon et al., 2014) and humpback whales (Apprill et al., 2011). The host-specificity of gut bacteriome may result from species loss and sorting due to the enrichment of nutrients, but can also result from active selection by the host, as indicated in the squid-*Vibrio* system (Kremer et al., 2013). Finally, physical and chemical barriers may limit competition and invasion of foreign microbes and therefore promote the local microbial colonization.

Interestingly, the pika gut bacterial community was more similar to plant than soil bacteriome. The results were consistent with those of Han et al. (2010), which showed the composition of gut bacteriome in grass carp was more similar to those in the ingested diet than to those in water and sediment (Han et al., 2010). In addition, our results showed that a majority of those core OTUs in pika gut were only sporadically observed in the plant bacteriome, indicating that those rare environmental bacteria are able to permanently retain once ingested. As diet-associated microbe reservoir, the plant-associated bacteria may partly colonize the guts. Thus, plant-associated bacteria are very important in regulating the bacterial community diversity of pikas.

### Pika gut may select for rare but diverse environmental bacteria

Our results showed that pika gut harbors bacteria that had generally a relative low abundance in the surrounding environment (plant or soil), as opposed to being colonized by bacteria that are enriched in the surrounding environment. Only minor overlap between the core bacteria of pikas and the most dominant environmental bacteria were observed. Notably, the relative abundances of shared OTUs by pikas and environment were negatively related. The same pattern has been observed in amphibian (Walke et al., 2014), crustacean (Mariadassou et al., 2015), sponge (Webster et al., 2010) systems. Diet and host factors influence the transmission and colonization by environmental microbes. In these systems, the skin or gut acts as a biological filter, and select for some certain members from free-living microbes in the environment. For pikas, there are at least three filter dynamics: (1) The plant constituents, such as cellulose and hemicellulose, may lead to the enrichment of microbes that degrade plant polysaccharides. (2) The plants produce various secondary compounds, such as tannin, which may result in a shift in the composition of gut bacteriome (Smith and Mackie, 2004). (3) Niche-selection in the digestive tract filters out species from the native pool that could not tolerate conditions in the gut environment. For example, the gastric juices have a very low pH, which exerts strong selection effects on gut microbial community (Beasley et al., 2015).

Notably, several core OTUs in pikas (two in Daurian pikas, three in plateau pikas) were not detected in the environment at all. This could be because these bacteria are rare in the environment, they were not detected with our current sequencing depth, or they are present in pikas' habitats other than the measured plant or soil, such as rare plant species in the steppe ecosystems. Another possible explanation is that these microbes are transmitted vertically from parents, or horizontally via conspecifics or individuals of different species. In general, pikas consume soft feces for protein utilization efficiently (Kizilova and Kravchenko, 2014), and feces-soil contamination is normal and frequent in the wild. Soil microbes may influence the assembly of pika gut bacterial communities. Thus, it is possible for the social behavior to aid the transmission of commensal microbes among animal individuals. We can hypothesize that the horizontal transmission should be more common than vertical transmission due to frequent transfer of symbionts from the environment to hosts under natural conditions. However, due to the complexity, diversity, and variability of host-associated symbionts, it is very difficult to evaluate transmission dynamics of these symbionts in wild environments.

In pika guts, the relative abundance of four predominant genera, including *Prevotella, Oscillospira, Ruminococcus, Treponema*, were also rare in the environment. Some members of the genera *Prevotella, Ruminococcus*, and *Treponema* harbor various cellulase and hemicellulase genes (Warnecke et al., 2007; Dai et al., 2014), and they are presumably involved in the degradation of plant polysaccharides in pika guts. Habitat filtering selects for specific bacteria, probably based on their key metabolic and functional potentials (Fierer et al., 2007; Philippot et al., 2010). This is further supported by the fact that the shared OTUs between pikas and environment were affiliated with diverse microbial taxa, implicating that host specific-selection in the digestive tract is not based on their phylogenetic traits, but most likely on their functional traits of specific microbial populations. Our results supported this inference, which showed that those shared microbes between pikas and environment were tightly linked with carbohydrate metabolism.

Community assembly comprises four distinct kinds of ecological processes, selection, drift, speciation and migration (Vellend, 2010), that all play important roles in the assembly of microbial communities (Costello et al., 2012). The plateau pikas and Daurian pikas had more similar microbial composition and structure than plant or soil bacteriome regardless of geographical locations or habitats. This suggests that selection plays a primary role in determining the assembly of gut bacteriome. In fact, although pika gut selects for those low-abundance environmental microbes, most of these rare environmental microbes were not dominant or core bacteria in pika guts, probably based on the flow of non-resident, transient bacteria associated with ingested food. Microbial assemblages are generally dominated by a small part of abundant taxa while most taxa are relatively rare, which is also consistent with the result of neutral assembly (Sloan et al., 2006). From the theory of meta-community dynamics, the assembly and maintenance of gut bacteriome may be the consequence of natural selection and neutral assembly process (Mariadassou et al., 2015).

In conclusion, by evaluating the bacterial community composition of wild pikas and the related environmental microbes from their habitats, we could demonstrate that the pika gut bacterial community showed manifest differences compared to environmental bacteria in the surrounding habitats. Notably, a small portion of low-abundance environmental bacteria are enriched in the pika gut. These rare microbes represent diverse bacterial taxa. Due to the host species-specificity, it is possible that guts of other wild mammals select for rare but diverse environmental microbes in each new generation. Thus, future work on a diverse assemblage of host gut bacterial communities and the related bacterial communities is needed to understand the microbial symbioses and transmission dynamics in the wild.

## Author contributions

HL designed research; HL, TL, MY, JL, SZ, and QL contributed to experimental work; HL finished the data analysis and wrote the manuscript. HL, XL, SW, and WC revised the manuscript.

### Conflict of interest statement

The authors declare that the research was conducted in the absence of any commercial or financial relationships that could be construed as a potential conflict of interest.
