# Supplementary figures and images for: Pika Gut May Select for Rare but Diverse Environmental Bacteria
Source: Front Microbiol. 2016 Aug 17;7:1269. doi: 10.3389/fmicb.2016.01269 (PMC4987353; doi:10.3389/fmicb.2016.01269)

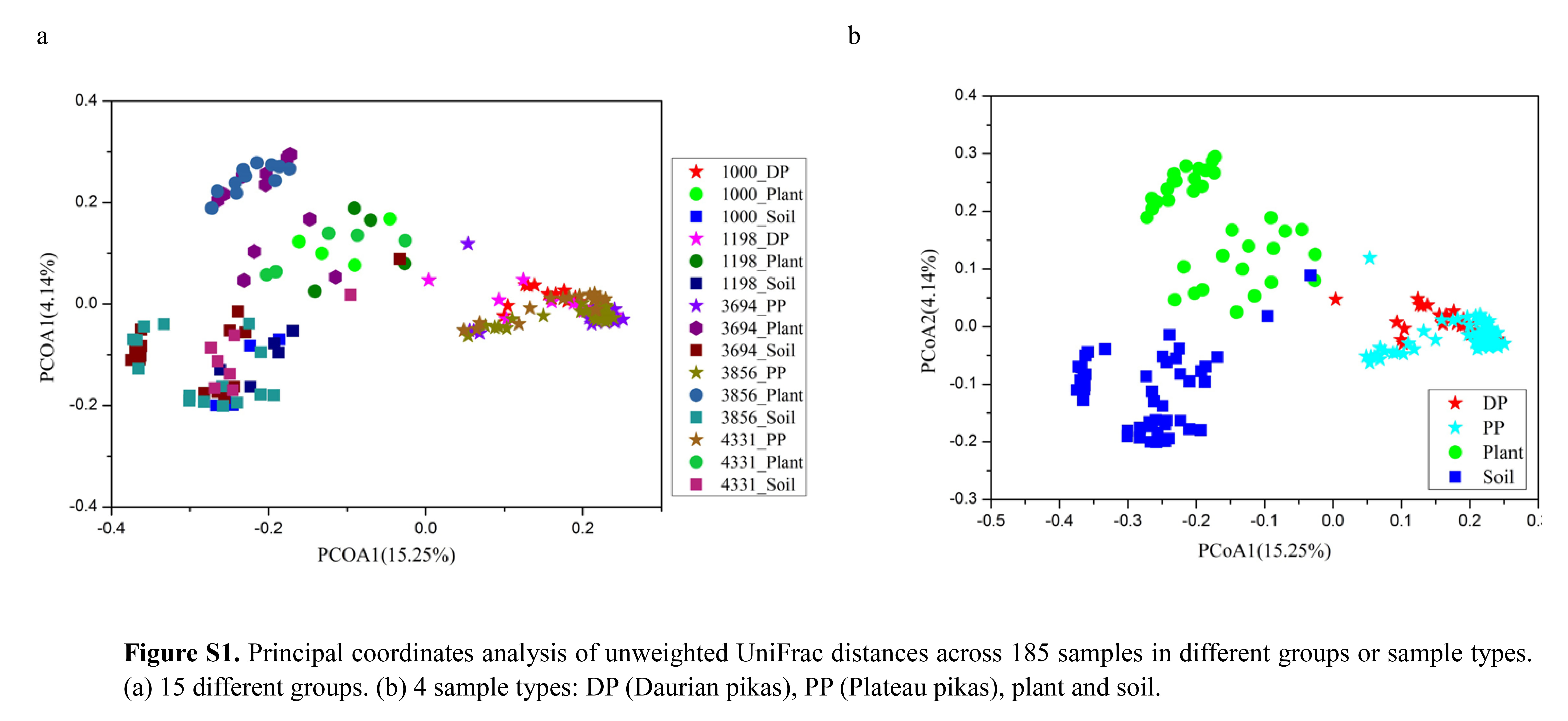

Supplement: Supplementary file 5 [file Image1.JPEG]

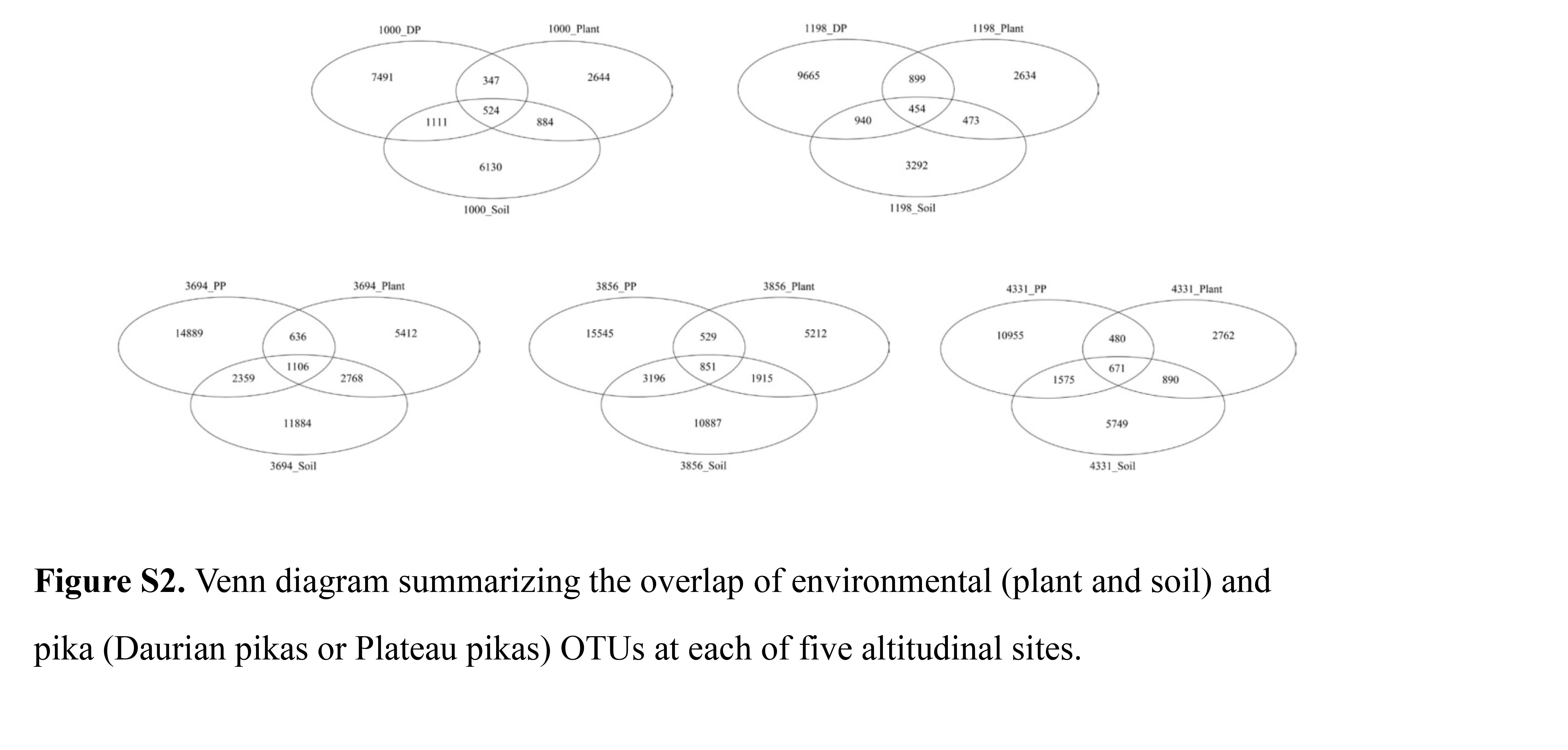

Supplement: Supplementary file 6 [file Image2.JPEG]

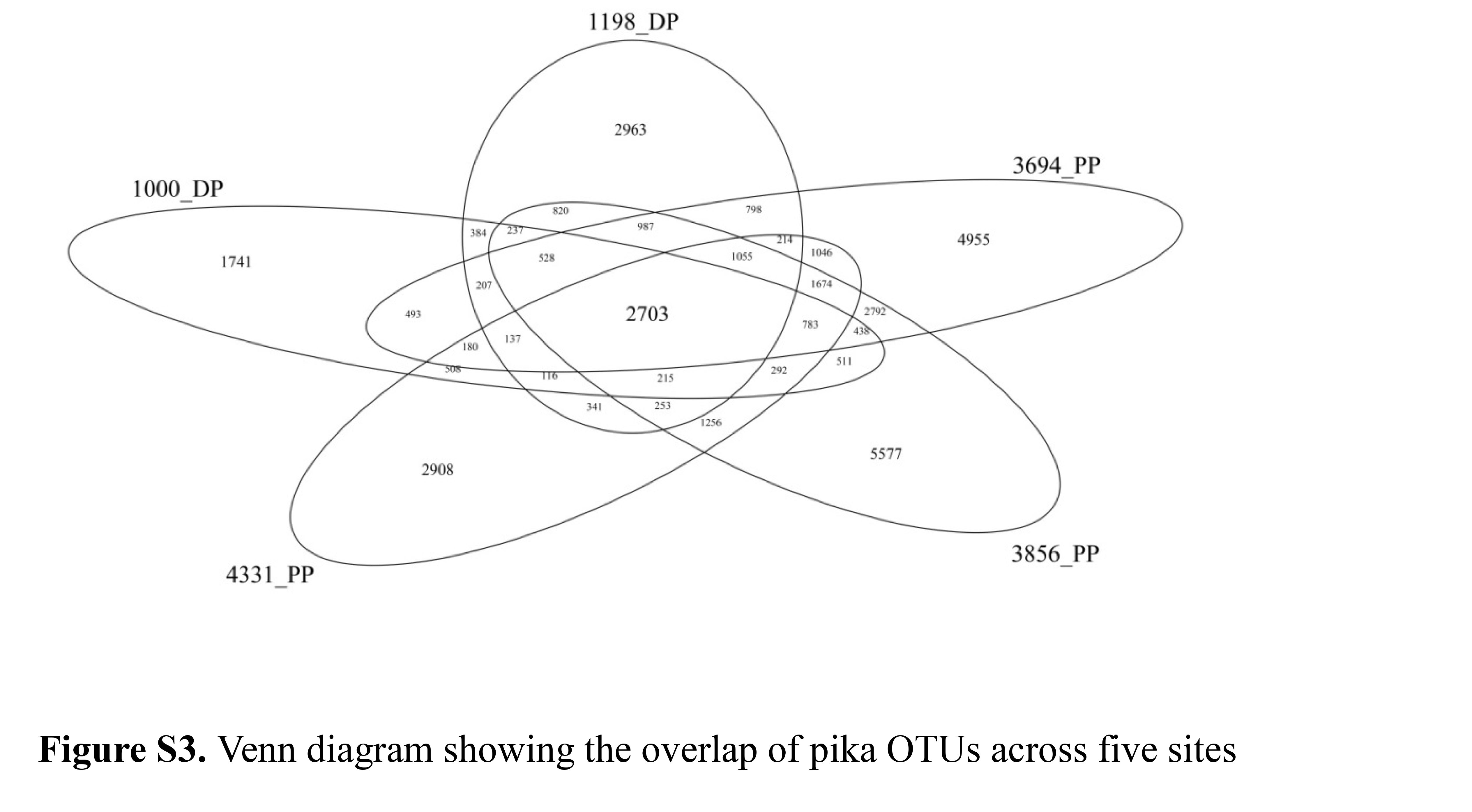

Supplement: Supplementary file 7 [file Image3.JPEG]

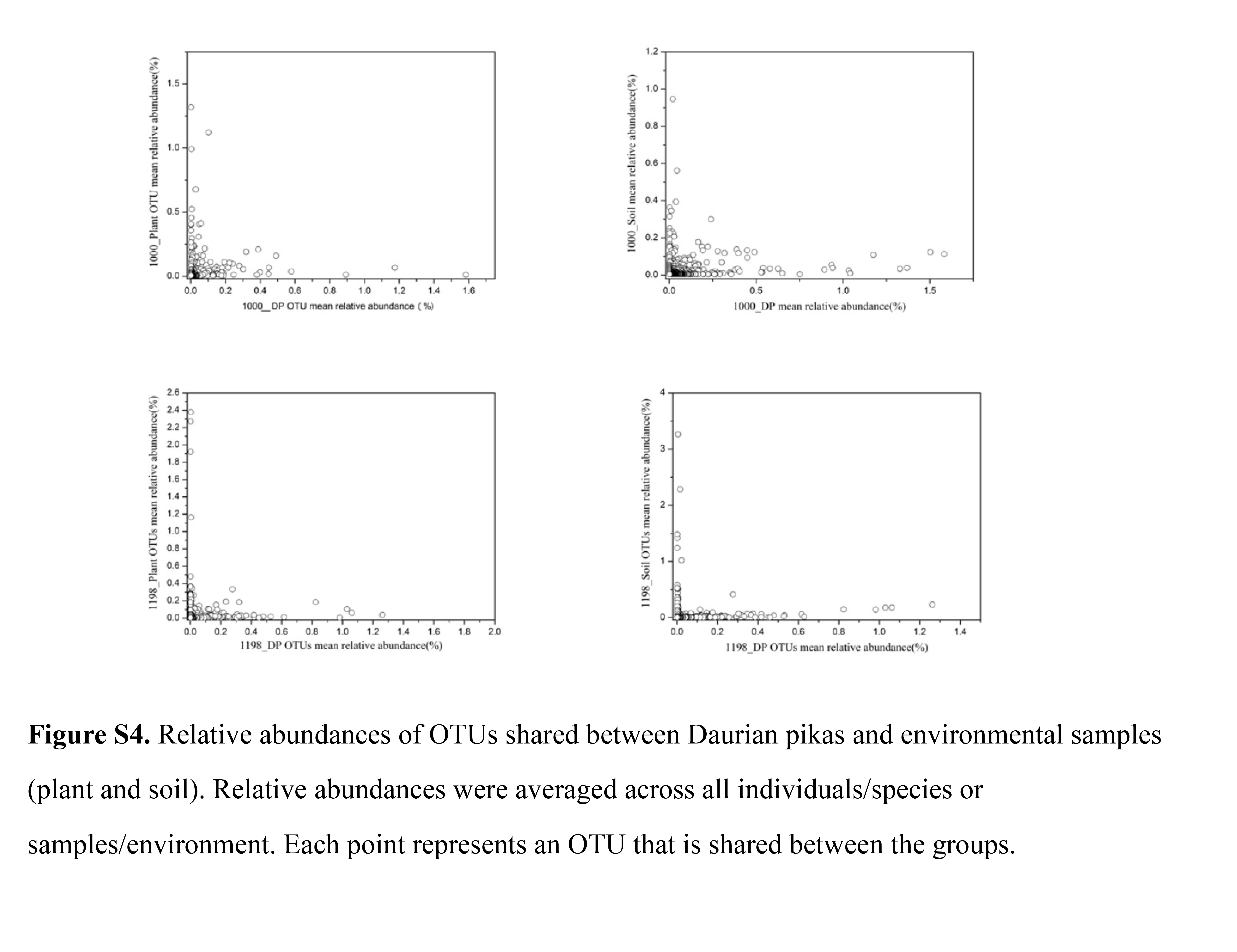

Supplement: Supplementary file 8 [file Image4.JPEG]

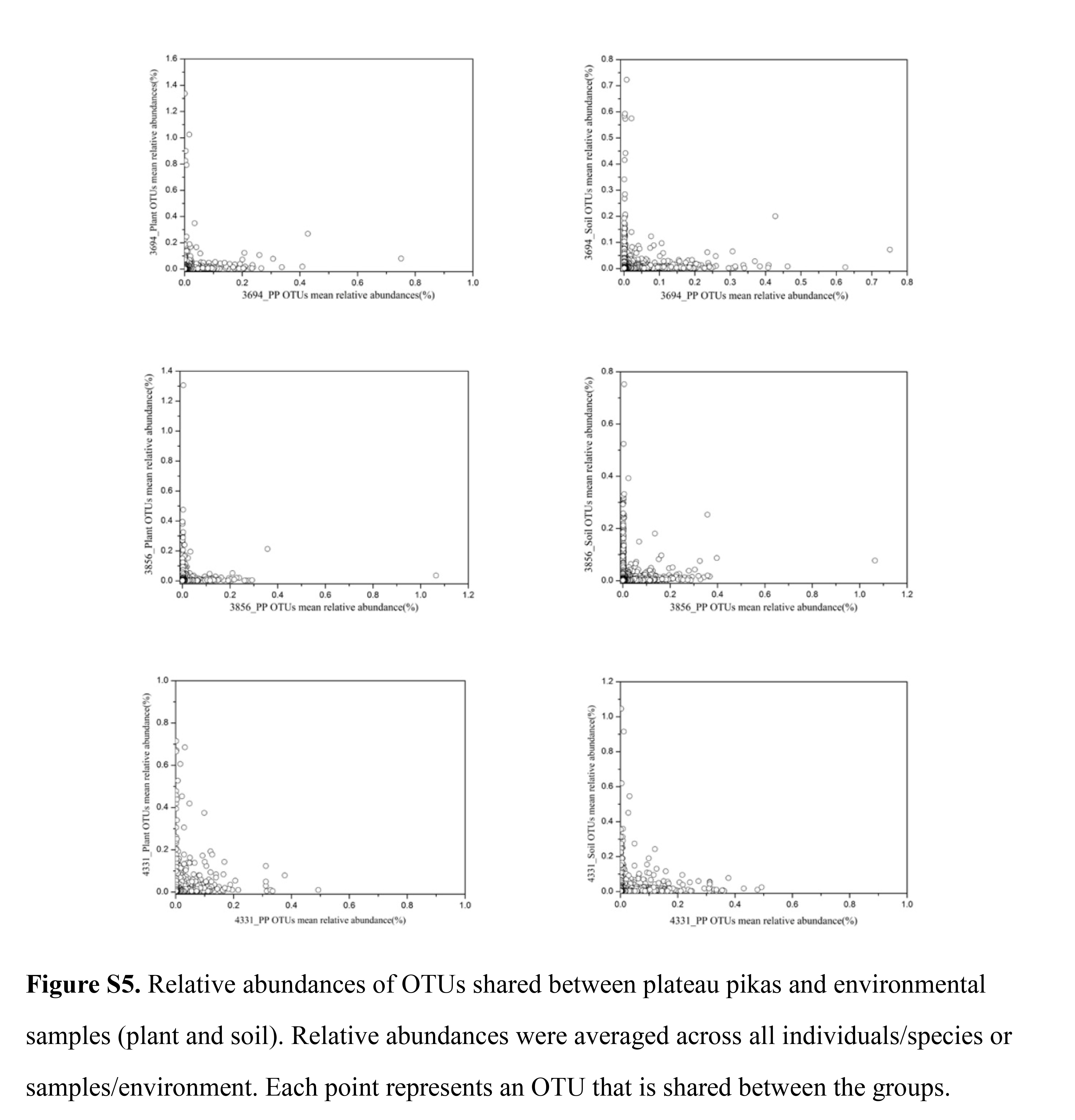

Supplement: Supplementary file 9 [file Image5.JPEG]

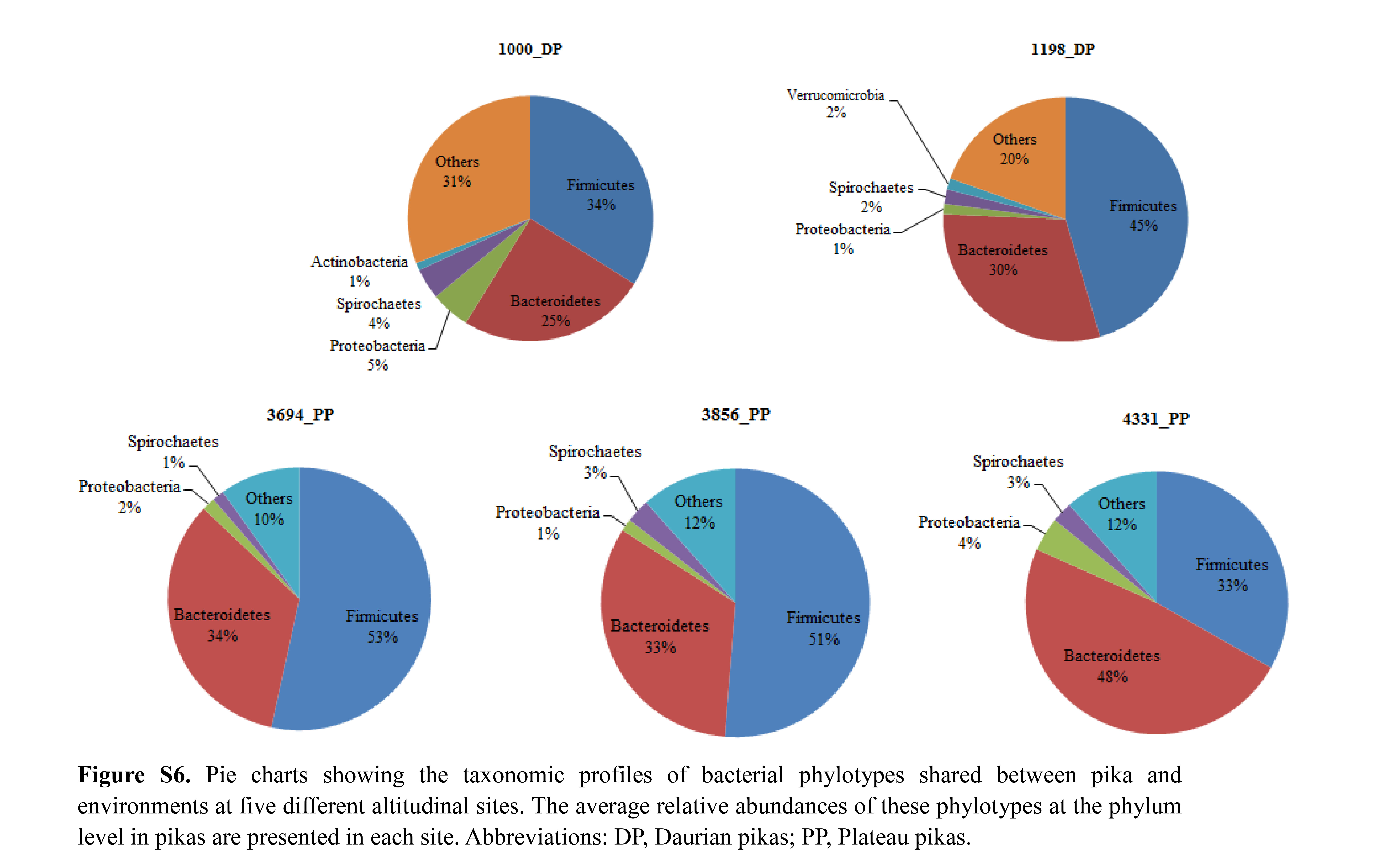

Supplement: Supplementary file 10 [file Image6.JPEG]

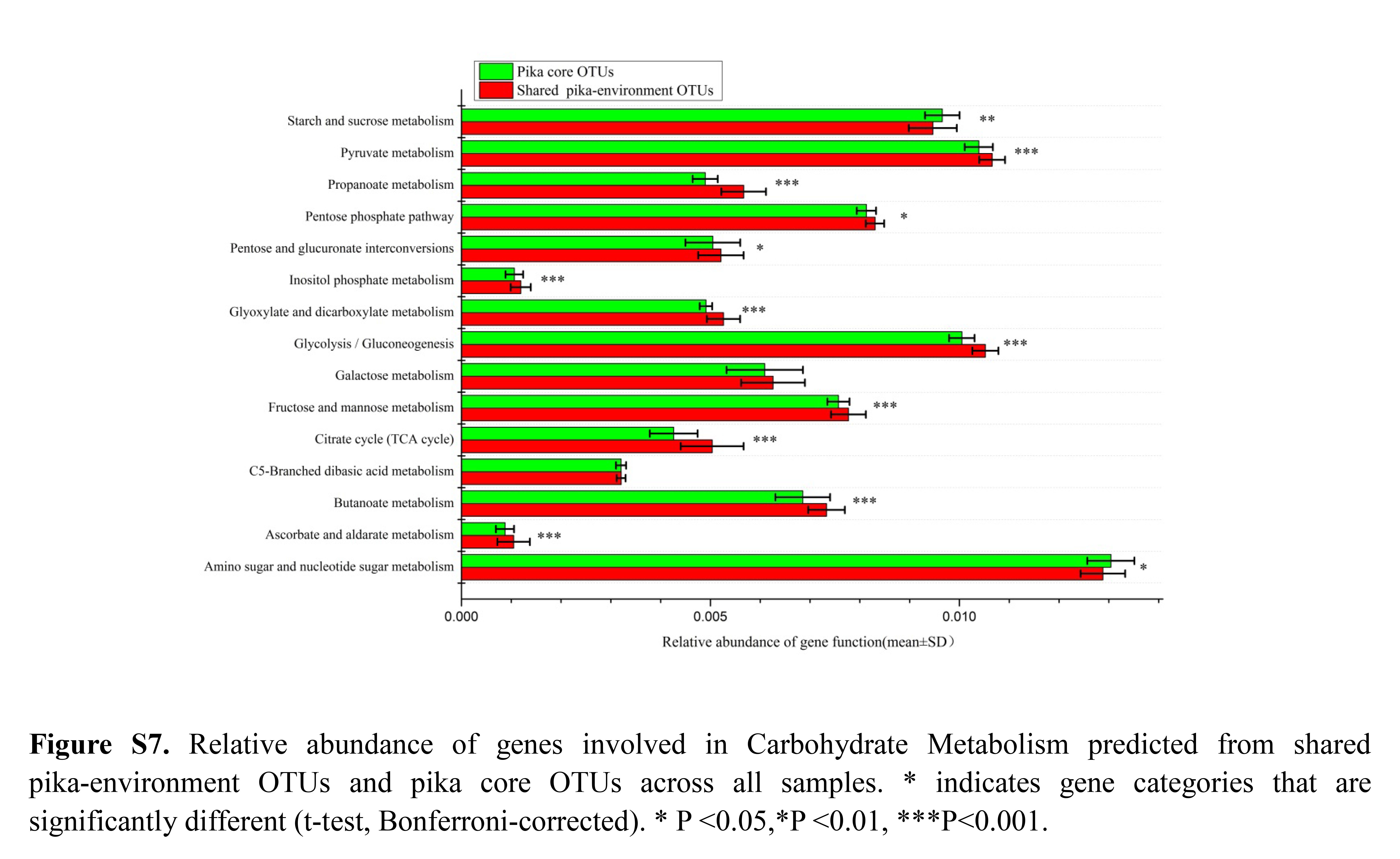

Supplement: Supplementary file 11 [file Image7.JPEG]
